# Quality and content evaluation of male HPV infection on Bilibili and TikTok: a cross-sectional study

**DOI:** 10.1038/s41598-026-54717-4

**Published:** 2026-05-28

**Authors:** Cheng Wang, Yuling Kou, Huanxin Sun, Zhonghua Li, Wei Kuang

**Affiliations:** 1 Department of Pathology, West China Second University Hospital of Sichuan University, Chengdu, Sichuan China; 2 Key Laboratory of Birth Defects and Related Diseases of Women and Children, Sichuan University, Chengdu, Sichuan China

**Keywords:** Male HPV infection, Health information, Short video, TikTok, Bilibili, Video quality, Diseases, Health care, Medical research

## Abstract

Social media platforms, particularly short-video applications, have emerged as crucial channels for disseminating public health information. Human papillomavirus (HPV) infection in males represents a significant sexually transmitted disease burden with under-addressed health impacts. Despite its prevalence, the quality of HPV-related content targeting male audiences on these platforms remains unevaluated, posing potential risks to public health literacy. We conducted a cross-sectional analysis of 265 TikTok and Bilibili videos addressing male HPV infection. Video reliability and educational quality were assessed using the modified DISCERN (mDISCERN) instrument and Global Quality Scale (GQS). The study used statistical analysis to examine the content and quality of videos on the two platforms, the identities of the uploaders and the correlations between these factors and user engagement data. Overall video quality was low (median GQS = 2.00; mDISCERN = 2.00). TikTok outperformed Bilibili in both engagement metrics and quality scores (GQS: TikTok median = 2.00 [IQR 2.00–3.00] vs. Bilibili = 2.00 [2.00–2.00], *p* < 0.01; mDISCERN: 2.00 [2.00–3.00] vs. 2.00 [2.00–2.00]). Content gaps were prominent: only 15.8% (*n* = 42) covered prevention strategies (e.g., vaccination), while symptom descriptions dominated (29.8%). Specialist-generated videos scored significantly higher in quality (GQS = 3.00 [2.00–3.00]; mDISCERN = 2.00 [2.00–3.00]) than non-specialist content (GQS = 2.00 [2.00–2.00]; mDISCERN = 2.00 [2.00–2.00]). Engagement metrics showed no correlation with quality scores. Short-video platforms exhibit suboptimal quality in disseminating male HPV information, with TikTok marginally superior to Bilibili. Specialist involvement enhances content reliability, underscoring the importance of leveraging professional health communication on social media. Public health initiatives must prioritise engaging experts to amplify accurate prevention messaging, particularly regarding vaccination, and address current informational inequities, thereby improving community health outcomes.

**Supplementary Information:**

The online version contains supplementary material available at 10.1038/s41598-026-54717-4.

## Introduction

Human papillomavirus (HPV) is one of the most common sexually transmitted infections^1^. Globally, 31.0% of males are infected with genital HPV^2^. In China, male HPV prevalence differs substantially across populations, reaching 52.45% in outpatients versus 7.89% in health-screening individuals^3^. HPV can infect the penis, scrotum, anus, mouth, and throat — particularly the oropharynx. Genital warts are the most common clinical manifestation of HPV in men^4^. Cancers associated with human papillomavirus (HPV) are the most serious health issues linked to HPV infection. Chronic infections play a significant role in the development of anal, oropharyngeal, and penile cancers in men^5^. The International Agency for Research on Cancer estimated that approximately 69,400 cases of cancer in men were caused by HPV in 2018^6^. The number of HPV-associated cancers in men is also increasing^7^. HPV infections in men are often overlooked due to the significant time lag between infection and diagnosis. Most infections are asymptomatic, and there are no routine screening methods tailored for men. This results in a large number of infections remaining undetected^8^. However, the potential impact of these infections on men’s health should not be underestimated. Additionally, male HPV infection can be transmitted to sexual partners and may affect fertility^9,10^. Therefore, it is vital to raise public awareness and understanding of HPV infection in men to improve public health.

As social media has developed rapidly, short video platforms have become a crucial means for people to access information and interact with one another^11,12^. As the two largest and most representative short-video platforms in China, TikTok and Bilibili reach the majority of users and complement each other by offering both short- and long-form content. This enables a comprehensive examination of the dissemination of health information^13–15^. While short videos are effective at conveying complex ideas, significant concerns exist regarding their content accuracy and reliability. The quality of health-related videos is highly variable, mainly due to the absence of rigorous peer review and regulatory oversight. This can lead to the inadvertent spread of misinformation about diseases and treatments, thereby distorting the public’s understanding of health practices^16,17^. The quality and dissemination of short-form video content about male HPV infection remain underexplored, especially in contrast to the substantial body of research on women^18–20^. Comparative investigations into how platforms like TikTok and Bilibili communicate such health information are virtually absent. This study, therefore, systematically analyzes male HPV-related short videos on these platforms to fill this critical research void.

This study systematically analyzed the content characteristics, information quality, engagement metrics, and uploader characteristics of short videos related to male HPV on TikTok and Bilibili. We aimed to clarify platform differences in content features and information quality, and to explore the current status of health information dissemination for men. The findings will help improve the quality of health-related content and provide evidence for platform governance and targeted health education.

## Materials and methods

### Video screening and data extraction

This study employed a cross-sectional design. As the search platforms only supported exact phrase matching during data extraction and our primary focus was HPV infection in men, we conducted searches on TikTok and Bilibili using the Chinese keyword “男性HPV感染” (male HPV infection) on November 5, 2025. To minimize the impact of personalized recommendation algorithms on the results, all searches were performed without logging in. The initial screening sample of the first 150 videos was selected based on each platform’s default “comprehensive ranking”.The exclusion criteria are as follows:

(1) Videos in languages other than Chinese were excluded; (2)Video content identified as advertising; (3) Videos unrelated to male HPV infection (those that do not directly involve topics such as epidemiology or transmission, or merely touch upon these topics superficially without providing any substantive information); (3) Videos uploaded within one week of the start of the data collection period, to ensure data stability and timeliness; (4) Content that is a duplicate or re-upload of material that has already been analyzed. Extract the following information from each video: platform source (TikTok/Bilibili), video duration (in seconds), engagement metrics (likes, comments, saves, and shares), and uploader characteristics.

### Uploader characteristics

The uploaders of the videos were categorized as (1) specialists, (2) non-specialists, and (3) individual users. Specialists were further divided into (1) dermatologists, (2) gynecologists, and (3) urologists. Non-specialists, including official social media accounts and specialists from other departments, such as the emergency department.

### Video quality and reliability assessment

The following two internationally recognized assessment tools, the mDISCERN scale and the Global Quality Score (GQS), are used to evaluate the quality and reliability of videos. Two authors (C. W. and W. K.) evaluated the quality of all the included videos independently. In cases of disagreement, a third reviewer acted as an adjudicator to reach a consensus. All participants have been informed and have consented to this study. GQS scoring utilizes a 5-point scale (1, extremely poor quality; 5, extremely high quality) to provide a comprehensive evaluation of the video’s overall quality, based on aspects such as professionalism, completeness of information, and clarity of expression^19^ (see Table S1 in the Supplementary Materials). The quality of information in short videos related to male HPV infection was evaluated using a 5-item mDISCERN tool adapted for digital health information. Each item was scored dichotomously as 1 (yes/adequate) or 0 (no/inadequate). To ensure reliability and reproducibility, operational definitions and scoring examples were developed for all items and are provided in the Supplementary Materials^21^ (see Table S2 in the Supplementary Materials). Additionally, the completeness of a video is assessed based on whether it includes information on the following topics: epidemiology, transmission, symptoms, diagnosis or screening, treatment, and prevention. Videos containing substantive health information were also evaluated based on the level of detail with which the uploader explained the relevant topic, with three possible ratings: no explanation (0 points), partial explanation (1 point), and complete explanation (2 points). Specialists, including dermatologists, gynecologists, and urologists (which include andrologists and urologists), differ from non-specialists, particularly those from other medical fields. All ratings were conducted by two evaluators who had relevant medical backgrounds. Before scoring, they received standardized training to ensure consistency in assessment criteria and minimize bias. In cases of significant discrepancies in scores, a third evaluator was consulted for arbitration.

### Statistical analysis

Data processing and analysis were performed using R version 4.3.3 (2024-02-29) and Zstats 1.0 (www.zstats.net). Descriptive statistics and non-parametric tests were employed to analyze video features and quality metrics. Categorical variables are presented as frequencies and percentages, and continuous variables are described using medians with interquartile ranges. We conducted a Shapiro–Wilk normality test and found that the group variables for the different platforms and uploaders were all non-normally distributed. The Mann–Whitney U test was used to compare engagement metrics and quality scores between TikTok and Bilibili. For multi-group comparisons, such as among specialists, non-specialists, and individual user videos, Kruskal–Wallis tests were applied. Spearman’s correlation analysis was used to explore the relationships between the GQS and the mDISCERN scores. The strength of the correlation was categorized as follows: Spearman *r* < 0.2 (negligible), 0.2–0.4 (weak), 0.4–0.6 (moderate), 0.6–0.8 (strong), and > 0.8 (very strong). A p-value of less than 0.05 was considered statistically significant.

## Results

### Video characteristics

**Fig. 1:**
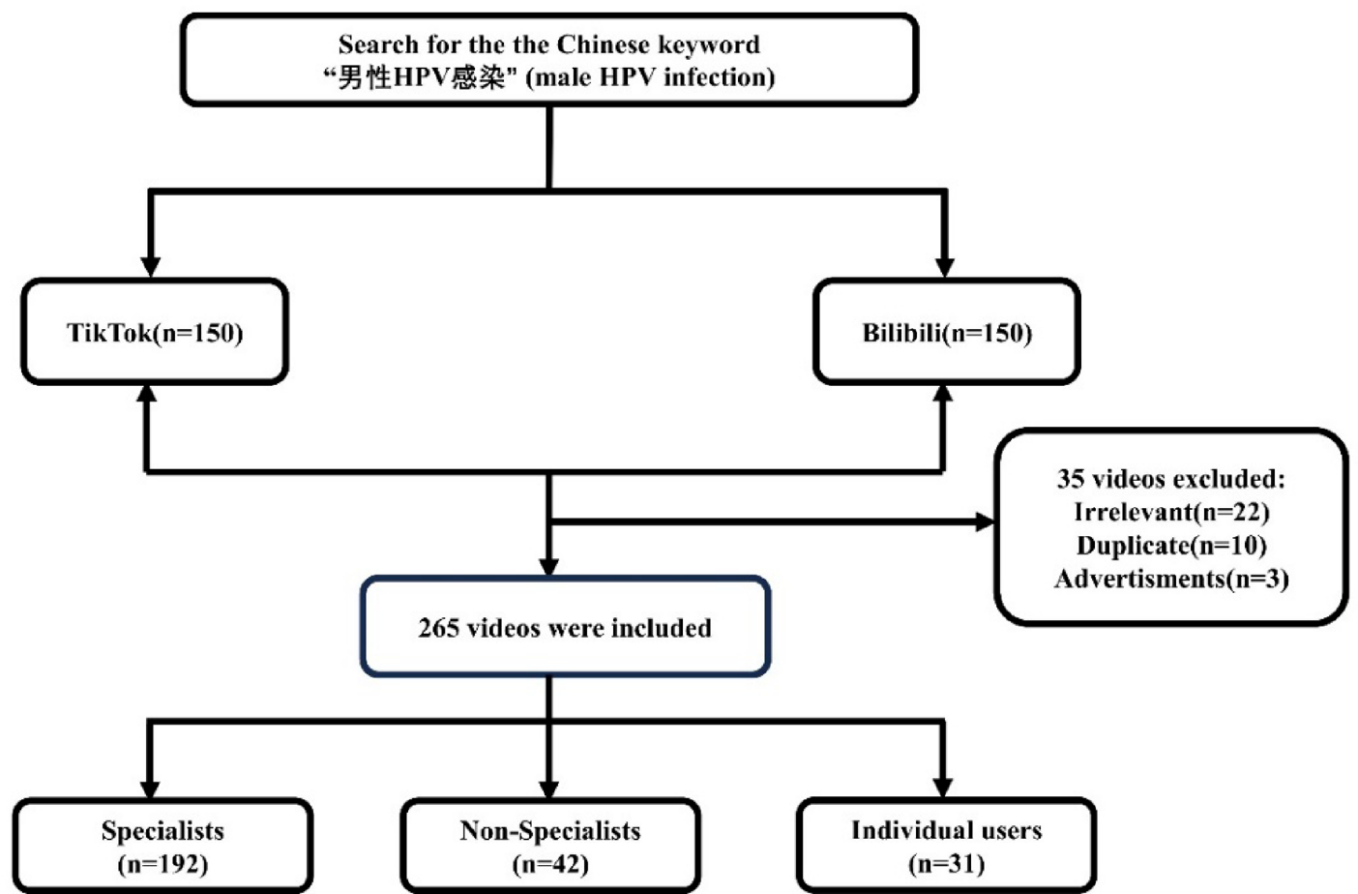
Study flowchart illustrating the video search strategy and screening process.

Systematic searches using the keyword “Male HPV Infection” in Chinese on TikTok and Bilibili initially identified a pool of videos, which were then refined through a rigorous filtering process. After excluding 22 irrelevant videos, 10 duplicate videos, and 3 advertisements, 265 videos remained for analysis, comprising 129 from TikTok and 136 from Bilibili. The detailed filtering process is illustrated in Fig. 1, while Table 1 presents the characteristics of the included videos. Overall, the video showed modest engagement metrics. The median Video lengths, likes, comments, collections, and shares per video were 52.00 (37.00, 78.00), 128.00 (3.00, 822.00), 22.00 (1.00, 198.00), 18.50 (0.00, 195.75), and 28.00 (1.00, 274.00). In terms of video quality, the median GQS score was 2.00 (2.00, 2.00), and the median mDISCERN score was also 2.00 (2.00, 2.00).

**Table 1:** Video characteristics. GQS: Global Quality Score; M, median; IQR: interquartile range.

Variables	Total (*n* = 265)
Short-video sharing platforms	
Tiktok	129(48.68%)
Bilibili	136(51.32%)
Video source [n (%)]	
Specialists	192(72.45%)
Non-Specialists	42(15.85%)
Individual user	31(11.70%)
General information	
Video length(s), M (IQR)	52.00 (37.00, 78.00)
Likes, M (IQR)	128.00 (3.00, 822.00)
Collections, M (IQR)	22.00 (1.00, 198.00)
Comments, M (IQR)	18.50 (0.00, 195.75)
Shares, M (IQR)	28.00 (1.00, 274.00)
Video quality	
GQS score, M (IQR)	2.00 (2.00, 2.00)
mDISCERN score, M (IQR)	2.00 (2.00, 2.00)

### Uploader characteristics

**Fig. 2:**
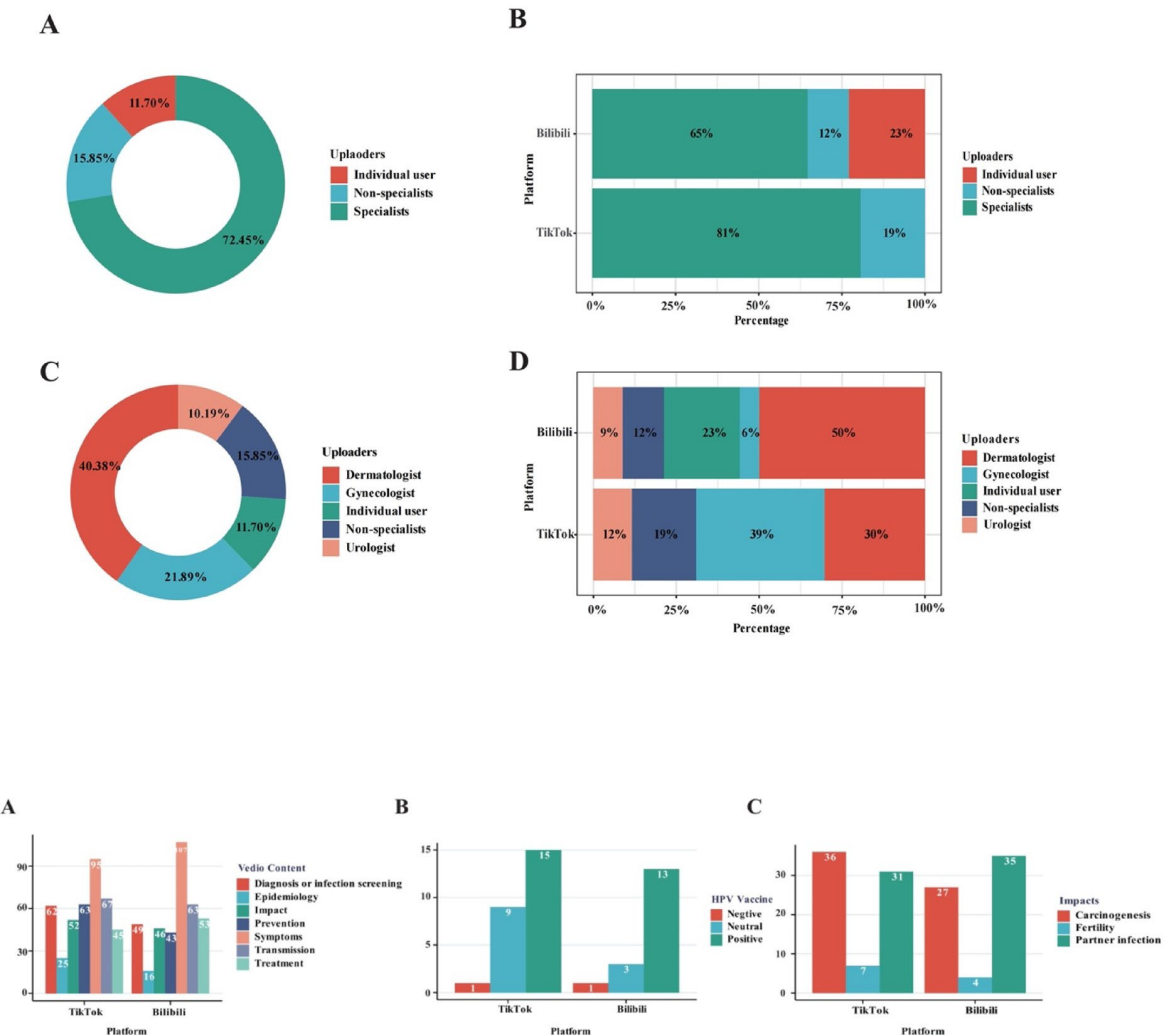
Distribution of video uploaders across platforms. (**A**) Overall uploaders’ composition. (**B**) Platform-specific comparison of uploader categories. (**C**) Detailed distribution of specialist subtypes (dermatologists, gynecologists, urologists), non-specialists, and individual users. (**D**) Platform-specific distribution of the detailed uploader categories.

In this study, Video sources are categorized into three groups: specialists (*n* = 192, 72.45%), non-specialists (*n* = 42, 15.85%), and individual users (*n* = 31, 11.7%) (Fig. 2A). Specialists were primarily classified as dermatologists (*n* = 107, 40.38%), gynecologists (*n* = 58, 21.89%), and urologists (*n* = 27, 10.19%), as shown in Table 1; Fig. 2C. On Bilibili, dermatologists dominated with 50% of uploads, followed by gynecologists (23%), whereas urologists, non-specialists, and individual users had lower shares. However, on the TikTok platform, gynecologists and dermatologists are the primary video uploaders, accounting for 39% and 30% respectively, with no uploads from individual users (as shown in Fig. 2B and D).

### Video content

**Fig. 3:**
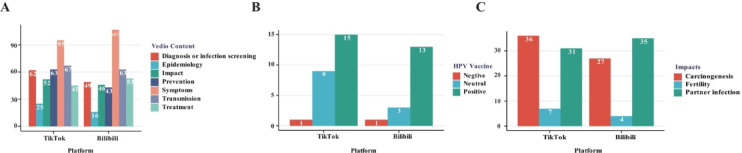
Comparative analysis of video content themes related to male HPV infection. (**A**) Distribution of core content topics. (**B**) Attitudes Toward the HPV Vaccine. (**C**) Discussion of health impacts.

Symptoms were the most frequently discussed topic, with 29.81% (*n* = 79) of videos providing detailed explanations. After that were impact and prevention, which accounted for 15.09% (*n* = 40) and 9.43% (*n* = 25) of the videos, respectively. Detailed information on epidemiology and diagnosis/screening for male HPV infection was rarely included, with only a few videos briefly mentioning these aspects (Table 2; Figure. 3A). Among the prevention videos, 42 mentioned HPV vaccination, with 25 on TikTok and 17 on Bilibili. Most of these videos provided positive recommendations for vaccination, with TikTok (*n* = 15) and Bilibili (*n* = 13) being the most prominent platforms (Fig. 3B). Of the videos addressing the impact of male HPV infection, 98 were mentioned, with 52 on TikTok and 46 on Bilibili. The most frequently mentioned impacts were carcinogenesis and partner transmission, followed by impacts on fertility. On TikTok, HPV’s carcinogenicity was the most commonly mentioned impact (*n* = 36), while on Bilibili, partner transmission was the most frequently mentioned risk (*n* = 35) (Fig. 3C).

**Table 2:** Completeness of video content.

Variables	Not involved (0 points)	Partial explanation (1 point)	Full explanation (2 points)
Epidemiology, n(%)	224 (84.53)	40 (15.09)	1 (0.38)
Transmission, n(%)	135 (50.94)	113 (42.64)	17 (6.42)
Symptoms, n(%)	63 (23.77)	123 (46.42)	79 (29.81)
Diagnosis or infection screening, n(%)	154 (58.11)	99 (37.36)	12 (4.53)
Treatment, n(%)	167 (63.02)	82 (30.94)	16 (6.04)
Prevention, n(%)	159 (60.00)	81 (30.57)	25 (9.43)
Impact, n(%)	167 (63.02)	58 (21.89)	40 (15.09)

**Fig. 4:**
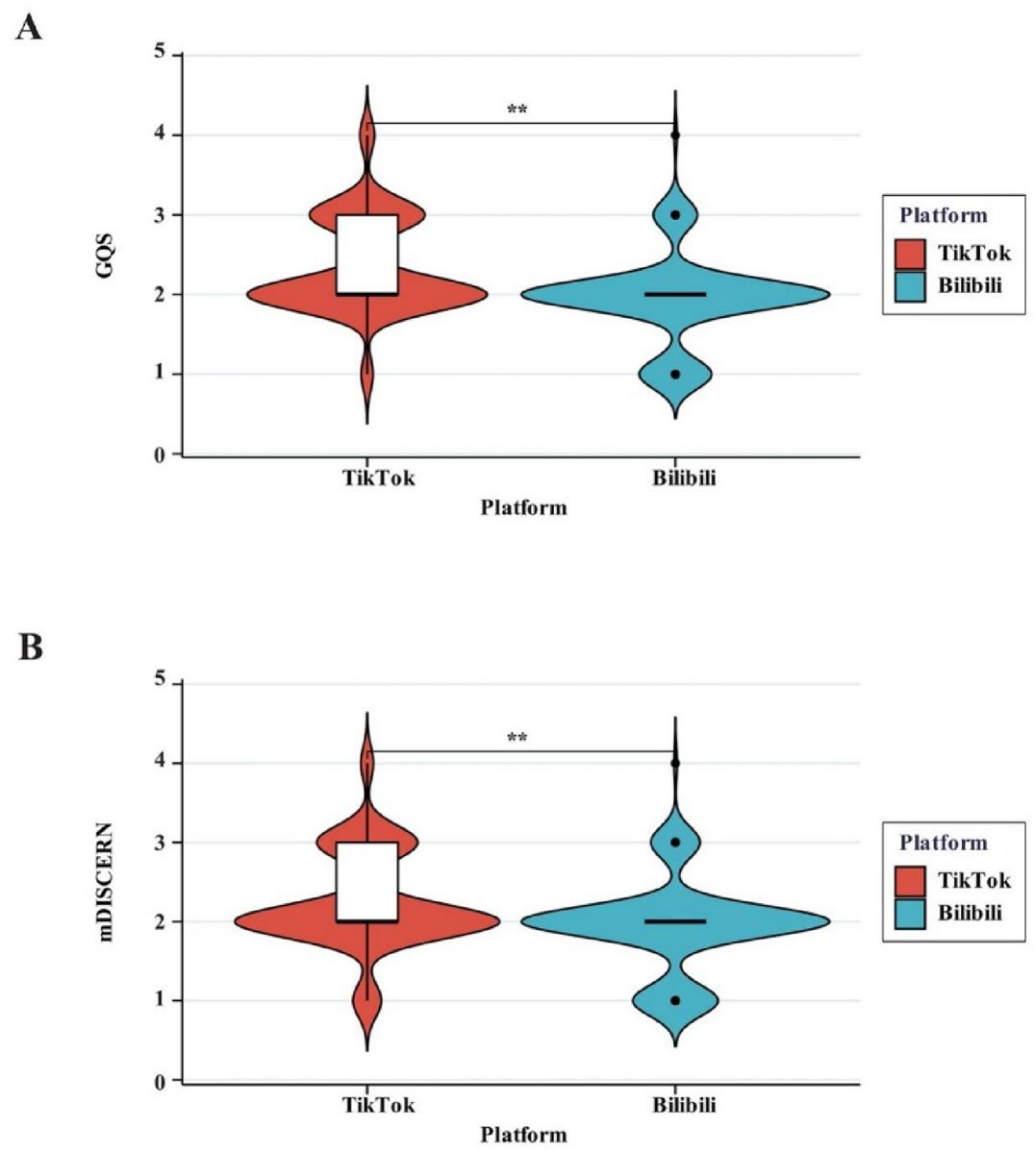
Comparative assessment of video quality and reliability between both platforms (TikTok and Bilibili) using standardized instruments. (**A**) Global Quality Scale (GQS) scores. (**B**) modified DISCERN (mDISCERN) scores. **, *p* < 0.01.

### Comparison of features across platforms

**Table 3:** provides a detailed comparison of video characteristics and quality between TikTok and Bilibili. Except for no statistically significant difference in video length, both platforms showed substantial differences in engagement metrics (*p* < 0.001). Engagement metrics differed significantly. TikTok videos demonstrated stronger audience interaction, receiving 719.00 likes (348.00, 2426.00), 136.00 saves (48.00, 817.00), 168.00 comments (49.00, 415.00), and 274.00 shares (79.00, 1555.00), while Bilibili videos scored lower across all engagement metrics (Table 3). Further comparative analysis showed that information quality differed significantly by platforms. TikTok videos scored significantly higher than Bilibili videos on GQS and mDISCERN, with values of 2.00 (2.00, 3.00) and 2.00 (2.00, 3.00), respectively, representing statistically significant differences (Fig. 4).

Variables	Bilibili (*n* = 136)	TikTok (*n* = 129)	*P*
General information			
Video length(s), M (IQR)	52.00 (33.75, 75.50)	53.00 (40.00, 81.00)	0.334
Likes, M (IQR)	3.00 (1.00, 16.75)	719.00 (348.00, 2426.00)	< 0.001
Collections, M (IQR)	1.00 (0.00, 8.25)	136.00 (48.00, 817.00)	< 0.001
Comments, M (IQR)	0.00 (0.00, 3.00)	168.00 (49.00, 415.00)	< 0.001
Shares, M (IQR)	1.00 (0.00, 7.00)	274.00 (79.00, 1555.00)	< 0.001
Quality and reliability			
GQS score, M (IQR)	2.00 (2.00, 2.00)	2.00 (2.00, 3.00)	< 0.001
mDISCERN score, M (IQR)	2.00 (2.00, 2.00)	2.00 (2.00, 3.00)	< 0.001

Table 3. General Information, Quality, and Reliability Scores of Male HPV Infection Videos on TikTok and Bilibili. GQS: Global Quality Score; M, median; IQR: interquartile range.

### Comparison of features across different uploaders

**Fig. 5:**
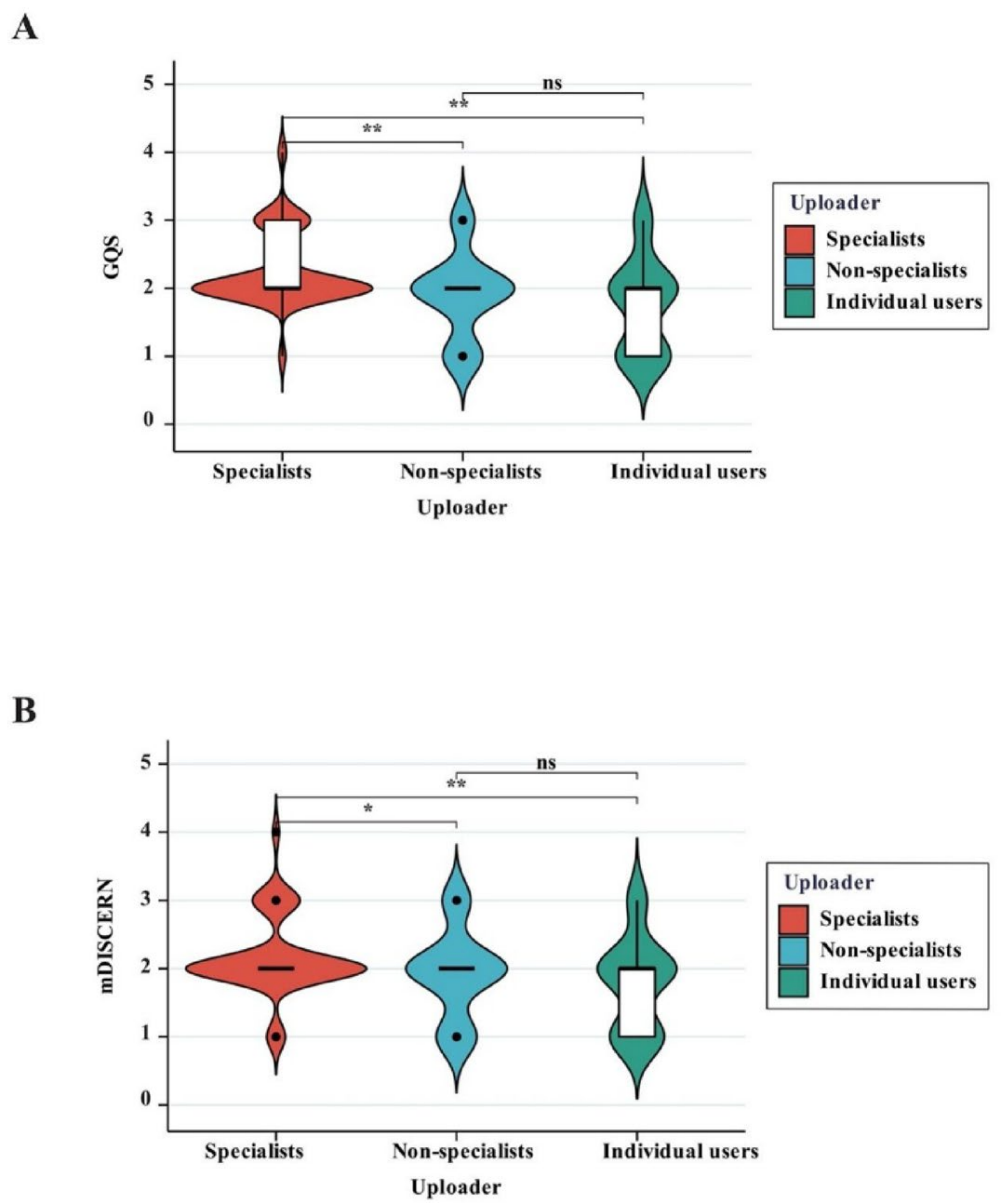
Comparison of quality and reliability scores across broad uploader categories. (**A**) GQS scores. (**B**) mDISCERN scores. *, *p* < 0.05; **, *p* < 0.01. ns, no statistical significance.

We also examined the effect of the video source on its characteristics and quality. In terms of popularity, videos uploaded by non-specialists had superior metrics for video duration, likes, comments, connections, and shares. In contrast, those uploaded by individual users had the poorest metrics for these interactions (*P* < 0.05) (Table 4). Information quality differed by uploader type: specialists produced higher-quality videos, whereas non-specialists and individual users frequently omitted sources or areas of uncertainty. The GQS and mDISCERN scores for the videos uploaded by specialists were 2 (2.3) and 2 (2.2), respectively. No significant differences were observed between videos uploaded by non-specialists and those uploaded by individual users (Fig. 5A and B). When specialists were further categorized into dermatologists, gynecologists, and urologists, videos uploaded by gynecologists received higher numbers of likes, comments, saves, and shares than those by dermatologists and urologists, except for video duration (Table S4). Regarding video quality, no statistically significant differences were observed among dermatologists, gynecologists, and urologists in GQS scores. For mDISCERN scores, no statistically significant differences were observed between dermatologists and gynecologists (Figure S1). The distribution and discrepancies in GQS and mDISCERN scores for each group are depicted in Figure S2.

**Table 4:** Comparison of different video sources. GQS: Global Quality Score; M, median; IQR: interquartile range.

Variables	Specialists (*n* = 192)	Non-Specialists(*n* = 42)	Individual users (*n* = 31)	*P*
Video length(s), M (IQR)	50.50 (35.00,71.25)	58.00 (43.25,94.25)	53.00 (39.00,114.00)	0.015
Likes, M (IQR)	138.50 (3.00,894.00)	362.50 (40.00,1003.00)	9.00 (1.00,63.00)	0.007
Collections, M (IQR)	25.00 (1.00,230.00)	74.00 (8.50,220.25)	4.00 (0.50,21.50)	0.029
Comments, M (IQR)	19.50 (0.00,202.00)	72.50 (8.25,230.25)	0.00 (0.00,12.50)	0.001
Shares, M (IQR)	30.00 (1.00,308.25)	139.50 (8.50,371.75)	4.00 (0.00,18.50)	0.008
GQS score, M (IQR)	2.00 (2.00,3.00)	2.00 (2.00,2.00)	2.00 (1.00,2.00)	< 0.001
mDISCERN score, M (IQR)	2.00 (2.00,2.00)	2.00 (2.00,2.00)	2.00 (1.00,2.00)	0.001

### Correlation analysis between video features and quality

**Fig. 6:**
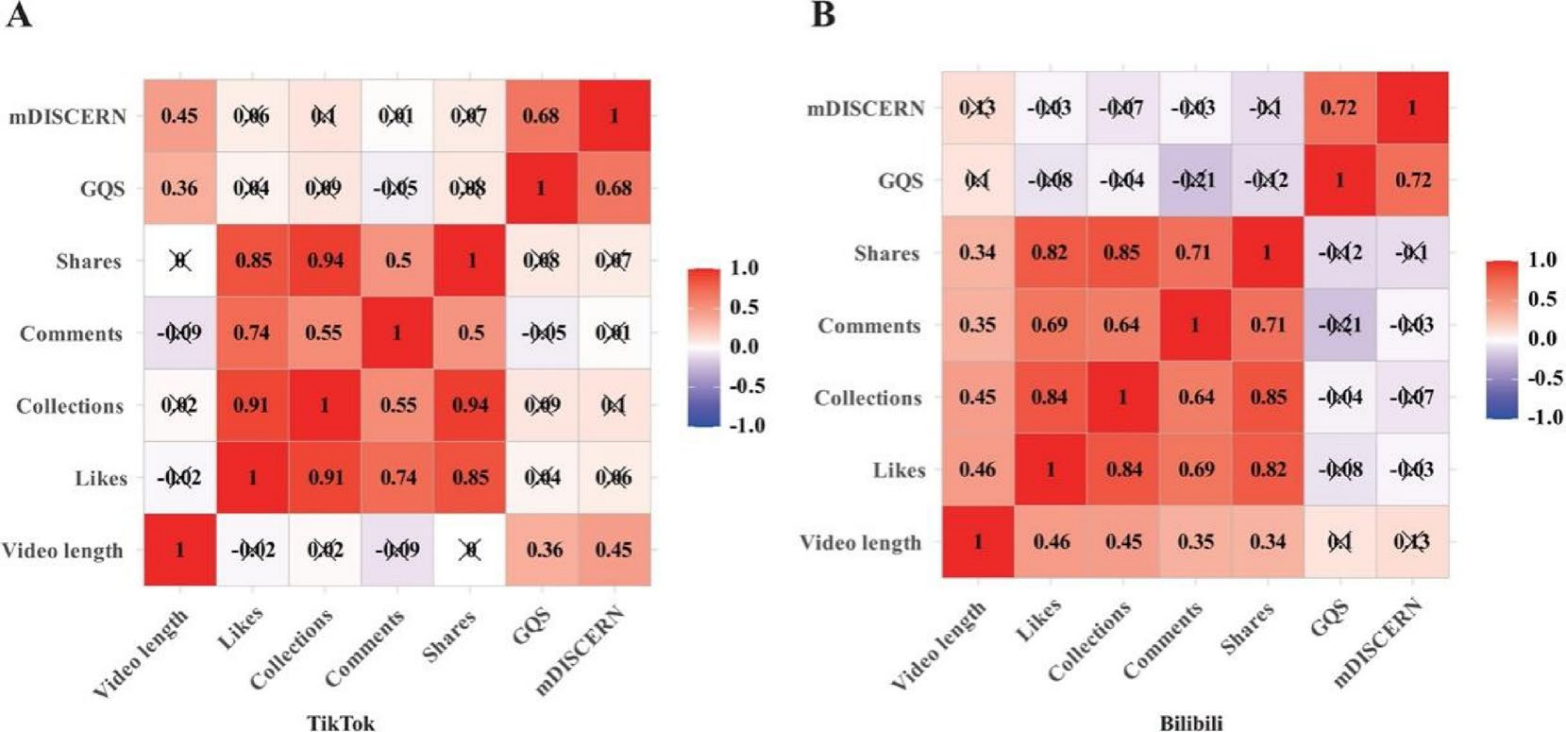
Spearman correlation matrix among key video variables, GQS, and mDISCERN scores.

Figure 6 presents the correlation analysis between video features and video quality on both TikTok and Bilibili platforms. Significant correlations were observed between GQS and the mDISCERN score on both platforms (*P* < 0.001). In contrast, no significant correlations were observed between interaction metrics (likes, comments, saves, shares) and quality scores in both platforms. On TikTok, video duration was moderately positively correlated with both GQS and the mDISCERN score (*P* < 0.001). On Bilibili, significant correlations were found between video duration, likes, comments, saves, and shares (*P* < 0.001).

## Discussion

The rapid growth of short-video platforms has established social media as a significant source of health information for the public, as two of the leading platforms in China’s short-video sector, TikTok and Bilibili, must be evaluated carefully to ensure that the health content they host is of high quality and reliable^12,22^. To date, no studies have examined content related to male HPV infection on these platforms. The video interaction metrics, GQS, and mDISCERN scores evaluated in this study suggest that user engagement and video quality on the TikTok platform are significantly higher than on Bilibili. An analysis of the video content reveals that coverage of each topic varies. Topics related to symptoms account for nearly 30% of the video content. Meanwhile, there are relatively few videos on HPV vaccination for men (only 42), most of which support the vaccination. A key finding was that content produced by medical specialists was of significantly higher quality than that from non-specialist doctors or individual users.

In terms of video content distribution, our analysis indicates that content creators on both platforms predominantly focused on presenting information about HPV symptoms in a clear and accessible manner. In contrast, discussions concerning the long-term health impacts and preventive measures received comparatively less attention. This observation aligns with findings from prior studies on health communication via social media, which frequently highlight a tendency to prioritize immediate, recognizable concerns over complex risk management and prevention strategies^11,23^. Topics that are less often discussed tend to be more specialized, which conflicts with the requirement for simplicity and accessibility in science communication. Although 42 videos mentioned HPV vaccination as a preventive measure, information on the vaccine’s specific benefits and its impact on public health remains insufficient. This may be due to the recent approval of the male HPV vaccine in China^24^. Therefore, public awareness of HPV infection in men still needs to be strengthened, particularly concerning the importance of vaccination^25,26^. While content on both platforms consistently highlighted the carcinogenic potential of HPV in men and its transmissibility to partners, its potential impact on male fertility was notably absent from most discussions. This disparity likely mirrors current gaps in public awareness. However, given that the influence of HPV on male reproductive health is increasingly recognized as a significant research frontier, this omission underscores a critical gap in public health messaging that warrants attention^27^.

A comparative analysis of TikTok and Bilibili reveals how distinct platform architectures, source demographics, and engagement mechanisms correlate with the dissemination of health information. Engagement metrics differed significantly: TikTok videos achieved higher average views, likes, comments, and shares than Bilibili videos. This may reflect TikTok’s short vertical format and algorithmic recommendation system, which are optimized for user engagement and reach^28^. By contrast, Bilibili’s community-focused structure and longer content culture limit comparable interactivity, illustrating the profound impact of platform ecology on engagement^29^. Furthermore, a notable paradox emerged: videos by non-specialist doctors, despite receiving lower quality scores, garnered significantly more likes, comments, and shares than those by specialists. This suggests that factors beyond clinical authority—such as compelling narrative style or perceived relatability—may drive public interaction more powerfully on these platforms.

However, when we assessed the quality of short videos related to HPV infection in men, we found that the majority were of poor quality, as indicated by the low total GQS and mDISCERN scores of 2.00 (2.00, 2.00). This finding is consistent with previous research^23,30,31^. Poor information quality does not imply factual errors. Videos may convey correct content yet remain poorly organized, incomplete, or lacking in transparency. Although specialists accounted for 72.5% of cases (192 out of 265), the overall quality remains unsatisfactory. No videos received a 5 score in the GQS score. This may be because most videos are relatively short, lack sufficient citations from authoritative sources, and are not very rigorous^32^. Furthermore, the variety of uploaders and the absence of regulatory review mechanisms on short video platforms have led to the frequent publication of low-quality and unreliable videos, as well as the dissemination of deceptive and misleading content to the public^33–35^. Quality assessments revealed that videos on TikTok received significantly higher scores on both the GQS and mDISCERN scales than those on Bilibili. Concurrently, the platforms differed markedly in their uploader profiles: individual users constituted 0% of sampled uploaders on TikTok compared to 23% on Bilibili. This user composition difference suggests that Bilibili’s content ecosystem is more substantially shaped by non-professional contributors, whose narratives may be more personal and subjective, potentially compromising informational reliability. Meanwhile, the superior quality among professional uploaders highlights the critical role of expertise in ensuring clear, well‑sourced, and responsible health communication. These findings collectively underscore the critical influence of the uploader’s background on content quality^11^. It also emphasizes the importance of expertise in disseminating health information, as high-quality content enhances public understanding and awareness of health issues^36^. Furthermore, while video quality scores did not differ significantly among specialist types, videos from gynecologists garnered considerably more likes and comments than those from dermatologists or urologists. This disparity in engagement may stem from the current knowledge framework: public information on male HPV often extrapolates from the extensive research on female HPV and cervical cancer, an area where gynecologists are perceived as primary authorities. This suggests that, even among experts, perceived relevance and topical authority influence audience engagement. To enhance both the perceived authority and the specialist diversity of content, future health communication initiatives should actively encourage participation from a broader range of domain experts, particularly andrologists and urologists, whose clinical focus aligns directly with male health.

The study also employed correlation analysis to investigate the relationship between video features and quality, revealing a significant correlation between GQS and mDISCERN. This suggests that video quality scoring systems are crucial for evaluating the scientific rigor and effectiveness of medical videos^14,37^. Meanwhile, a moderate positive correlation was observed between video length and quality on TikTok, but no significant correlation was found on Bilibili. This discrepancy may indicate differing user preferences regarding video duration across platforms, suggesting that creators should consider audience preferences when crafting content to optimize its dissemination^38^. Video likes, comments, saves, and shares are all unrelated to quality, consistent with previous research^39^.

Our findings offer clear implications for public health communication. Given TikTok’s higher engagement, public health authorities could develop platform-specific guidelines to improve clarity and the reliability of sources while maintaining audience appeal. In contrast, given the low proportion of expert creators on Bilibili, partnering with health professionals would help strengthen the authority of content and enhance overall quality. Indeed, since medical professionals consistently produce higher-quality videos, supporting their wider involvement could effectively improve public understanding, raise health awareness, and promote more informed health decisions among men. However, several limitations warrant caution. First, the sampling strategy, limited by specific keywords and timeframes, may not capture the whole content universe, potentially affecting generalizability^33,40^. To mitigate this limitation, future studies may combine diverse keyword sets and adopt iterative expansion based on pilot samples to enhance coverage and update search terms effectively. Second, the study design cannot assess the long-term impact of viewing such content. Third, although TikTok and Bilibili dominate the domestic market, other international short video platforms, such as Instagram Reels and YouTube Shorts, were excluded. This restricts the scope for cross-cultural comparisons. Future work should broaden the scope of the analysis by comparing it with global platforms such as YouTube and Instagram. It should also adopt a longitudinal research design to track the impact of information over time. Furthermore, excluded advertisement videos may retain some health information, and future research could assess their educational value via automated text analysis or broader inclusion criteria. In addition, low-quality videos in this study should be distinguished from misinformation; specialized coding schemes can be used to classify false information, thereby providing a clearer understanding of online health information regarding male HPV infection. Finally, without demographic data on viewers or creators (e.g., age, gender, location, education, income), this video-only analysis cannot assess whether the content reaches high-risk populations (e.g., sexually active or immunocompromised individuals, unvaccinated adolescents). Future research should include user data or surveys to validate content suitability and accessibility across target groups.

## Conclusion

In conclusion, our analysis of male HPV-related content on TikTok and Bilibili highlights the dual role of short-video platforms as both powerful tools for and significant challenges to effective health communication. While expert-generated content proved to be a key reliable resource, the overall information landscape was of concerning quality. The public must navigate this terrain with caution, critically evaluating sources. This necessitates that platforms themselves assume greater responsibility by structurally promoting content from credentialed health professionals, thereby safeguarding public health within the digital ecosystem.

## Supplementary Information

Below is the link to the electronic supplementary material.

Supplementary Material 1

## Data Availability

The original contributions presented in the study are included in the article/Supplementary material; further inquiries can be directed to the corresponding author.
