# Supplementary material for: Quality and content evaluation of male HPV infection on Bilibili and TikTok: a cross-sectional study
Source: Sci Rep. 2026 May 28;16:24447. doi: 10.1038/s41598-026-54717-4 (PMC13448700; doi:10.1038/s41598-026-54717-4)
Supplement: Supplementary file 1 [file 41598_2026_54717_MOESM1_ESM.docx]

Table S1 The Global Quality Score (GQS) quality criteria.

| Item features | Points |
| --- | --- |
| Poor quality; poor flow of the videos; most information missing; not at all useful for patients | 1 |
| Generally poor quality; some information listed, but many important topics are missing; of very limited use to patients | 2 |
| Moderate quality; suboptimal flow; some important adequately discussed, but other information poorly discussed; somewhat useful for patients | 3 |
| Good quality and generally good flow; most of the relevant information is listed, but some topics are not covered; useful for patients | 4 |
| Excellent quality and flow; very useful for patients | 5 |

Table S2 The Modified DISCERN (mDISCERN) quality criteria.

Reliability Score

1. Is the video clear, concise, and understandable?

2. Are valid sources cited?

3. Is the content presented balanced and unbiased?

4. Are additional sources of content listed for patient reference?

5. Are areas of uncertainty mentioned?

Table S3 Characteristics, Quality, and Reliability of Male HPV Infection Videos by Different Uploaders on TikTok and Bilibili.

| Variables | Dermatologists (n = 107) | Gynecologists(n = 58) | Urologist (n = 27) | Non-Specialists(n = 42) | Individual users (n = 31) | P |
| --- | --- | --- | --- | --- | --- | --- |
| Video length(s), M (IQR) | 53.00 (36.50,71.50) | 52.00 (35.25,86.00) | 45.00 (29.00,52.00) | 58.00 (43.25,94.25) | 53.00 (39.00,114.00) | 0.007 |
| Likes, M (IQR) | 15.00 (2.00,755.50) | 502.50 (174.00,1626.00) | 88.00 (1.50,589.50) | 362.50 (40.00,1003.00) | 9.00 (1.00,63.00) | <.001 |
| Collections,M (IQR) | 7.00 (1.00,220.00) | 78.50 (22.75,321.75) | 9.00 (0.00,100.00) | 74.00 (8.50,220.25) | 4.00 (0.50,21.50) | <.001 |
| Comments, M (IQR) | 2.00 (0.00,93.00) | 132.50 (29.00,394.25) | 12.00 (0.00,185.50) | 72.50 (8.25,230.25) | 0.00 (0.00,12.50) | <.001 |
| Shares, M (IQR) | 4.00 (0.00,243.50) | 125.50 (32.00,936.00) | 15.00 (0.00,286.50) | 139.50 (8.50,371.75) | 4.00 (0.00,18.50) | <.001 |
| GQS score,M (IQR) | 2.00 (2.00,2.00) | 2.00 (2.00,3.00) | 2.00 (2.00,2.00) | 2.00 (2.00,2.00) | 2.00 (1.00,2.00) | <.001 |
| mDISCERN score,M(IQR) | 2.00 (2.00,2.00) | 2.00 (2.00,3.00) | 2.00 (2.00,2.00) | 2.00 (2.00,2.00) | 2.00 (1.00,2.00) | 0.001 |

GQS: Global Quality Score; M, median; IQR: interquartile range

Details in Methods

Operational Definitions and Scoring Examples for the mDISCERN Instrument

General Coding Rules: Each item was scored dichotomously:

1 = Yes (present/adequate)

0 = No (absent/inadequate)

Two independent reviewers rated all videos. Discrepancies were resolved by discussion to achieve consensus. All definitions and examples were tailored to content about male HPV infection, transmission, associated diseases, testing, vaccination, and prevention.

1. Is the video clear, concise, and understandable?

Operational definition

The video uses plain language, avoids unexplained medical jargon, maintains logical flow, and clearly conveys key information about male HPV in a short‑video format.

Score 1 (Yes): The video clearly explains male HPV transmission, associated conditions (e.g., genital warts, anal/oropharyngeal cancer), and vaccination using simple, easy‑to‑follow language.

Score 0 (No):The video is confusing, overly technical, disorganized, or fails to clearly communicate meaningful information about male HPV.

2. Are valid sources cited?

Operational definition

The video explicitly references credible, authoritative sources such as WHO, CDC, national health authorities, clinical guidelines, or peer‑reviewed literature.

Score 1 (Yes):The video states “According to WHO guidelines…” or “Based on recent CDC recommendations for HPV vaccination in men.”

Score 0 (No):No source is provided; information relies solely on personal opinion or vague claims without attribution.

3. Is the content presented balanced and unbiased?

Operational definition

The video provides evidence‑based information without exaggeration, fearmongering, one‑sided emphasis, or misleading claims. It appropriately reflects that most HPV infections are asymptomatic and self-limited.

Score 1 (Yes):The video notes that most HPV infections in men resolve spontaneously, while also describing potential risks of persistent infection.

Score 0 (No): The content overstates risks, misrepresents facts, is heavily biased, or presents one-sided information without context.

4. Are additional sources of content listed for patient reference?

Operational definition

The video provides guidance for further information, such as official websites, healthcare consultations, or other reliable resources.

Score 1 (Yes):The video advises viewers to consult a physician or visit an official health website for more information on male HPV.

Score 0 (No): No additional resources or guidance for further reading or consultation is provided.

5. Are areas of uncertainty mentioned?

Operational definition

The video explicitly acknowledges uncertainties or knowledge gaps specific to male HPV, including:lack of routine screening guidelines, limited evidence on long-term outcomes of asymptomatic infection, inconsistent clinical recommendations, or insufficient population‑based data in men.

Score 1 (Yes):The video mentions that routine HPV screening is not widely established for men or that evidence for certain management strategies remains limited.

Score 0 (No):All claims are presented as definitive facts; no uncertainties, limitations, or knowledge gaps are acknowledged.
